# G196 epitope tag system: a novel monoclonal antibody, G196, recognizes the small, soluble peptide DLVPR with high affinity

**DOI:** 10.1038/srep43480

**Published:** 2017-03-07

**Authors:** Kasumi Tatsumi, Gyosuke Sakashita, Yuko Nariai, Kosuke Okazaki, Hiroaki Kato, Eiji Obayashi, Hisashi Yoshida, Kanako Sugiyama, Sam-Yong Park, Joji Sekine, Takeshi Urano

**Affiliations:** 1 Department of Biochemistry, Shimane University School of Medicine, Izumo 693-8501, Japan; 2 Department of Oral and Maxillofacial Surgery Shimane University School of Medicine, Izumo 693-8501, Japan; 3 Drug Design Group, Kanagawa Academy of Science and Technology, Kawasaki, Kanagawa, 213-0012, Japan; 4 Protein Design Laboratory, Graduate School of Medical Life Science, Yokohama City University, Tsurumi, Yokohama 230-0045, Japan

## Abstract

The recognition specificity of monoclonal antibodies (mAbs) has made mAbs among the most frequently used tools in both basic science research and in clinical diagnosis and therapies. Precise determination of the epitope allows the development of epitope tag systems to be used with recombinant proteins for various purposes. Here we describe a new family of tag derived from the epitope recognized by a highly specific mAb G196. The minimal epitope was identified as the five amino acid sequence Asp-Leu-Val-Pro-Arg. Permutation analysis was used to characterize the binding requirements of mAb G196, and the variable regions of the mAb G196 were identified and structurally analyzed by X-ray crystallography. Isothermal titration calorimetry revealed the high affinity (*K*_d_ = 1.25 nM) of the mAb G196/G196-epitope peptide interaction, and G196-tag was used to detect several recombinant cytosolic and nuclear proteins in human and yeast cells. mAb G196 is valuable for developing a new peptide tagging system for cell biology and biochemistry research.

Monoclonal antibodies (mAbs) are among the most frequently used tools in basic science research and in clinical diagnosis and therapies^1^,^2^,^3^. Identification of the target epitope is of critical importance in the characterization of a mAb. To this end, understanding antibody specificity at the amino acid level provides key information for understanding the specific interaction between antibodies and their epitopes. mAb/epitope pairs provide a powerful tool as anti-tag mAb/tags when specific antibodies for the protein of interest are not readily available. There are a broad range of applications of mAb/epitope pairs in experimental biology, ranging from human to yeast, including monitoring protein expression, tracking and localizing proteins at subcellular levels, protein purification, the analysis of protein topology, dynamics and interactions, and the analysis of structural and functional proteomics^4^,^5^,^6^,^7^. The most commonly used and well-characterized anti-tag mAb/tags are commercially available and include M2/FLAG-tag (DYKDDDDK)^8^, 9E10/c-Myc-tag (EQKLISEEDL)^9^, and 12CA5/HA-tag (YPYDVPDYA)^10^.

Short epitope tags (8–10 amino acid residues) are advantageous for minimizing side effects on the structure and biological function of the fused target protein^4^,^7^. However, each tag designed to date has unique disadvantages and all tags, whether large or small, occasionally interfere with the structure, biological activity and/or crystallization of the fused protein^11^,^12^,^13^,^14^,^15^. Furthermore, epitope tags are known to be post-translationally modified by phosphorylation, glycosylation, and sulfation in cells^16^,^17^, and these modifications increase the molecular mass of the fused protein and change gel mobility. In addition, it has been reported that epitope modification can abolish anti-tag mAb/tag recognition^17^.

In the present study, we generated a mAb, named G196, against glutathione *S*-transferase (GST) protein bacterially expressed using the pGEX-2T vector, and determined that the epitope of G196 corresponds to a sequence of five amino acids in the C-terminal extension of the protein encoded by pGEX-2T, and not to GST protein itself. The mAb G196/G196-epitope interaction was characterized by permutation analysis, isothermal titration calorimetry (ITC), and structural analysis. This mAb G196/G196-epitope suggests that it may be possible to generate a specific mAb and a corresponding new epitope tag.

## Results

### A novel mAb, named G196

**Figure 1:**
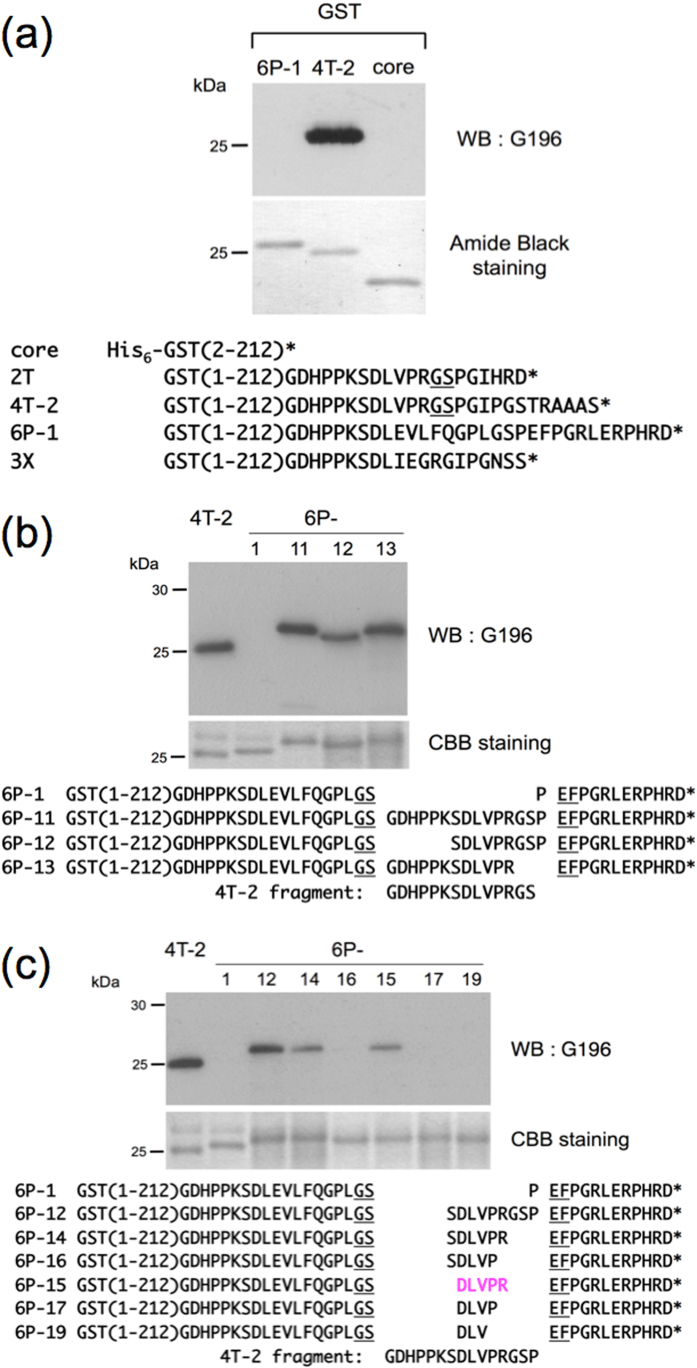
Epitope mapping of mAb G196. (**a**) Western blot (WB) analysis using mAb G196 (upper panel) and amide black staining (middle panel) of the purified bacterial proteins. Amino acid sequence alignment of the C-terminal extension of the proteins encoded by pGEX vectors (lower panel): 6P-1, the protein encoded by pGEX-6P-1; 4T-2, pGEX-4T-2; 2 T, pGEX-2T; 3X, pGEX-3X; core, GST(2–212). *Bam*HI restriction site of the pGEX-2T- or pGEX-4T-2-encoded Gly-Ser (GS, indicated with underlines). (**b**) and (**c**) Western blot analysis using G196 mAb (upper panel) and Coomassie Brilliant blue (CBB) staining (middle panel) of the bacterially expressed proteins shown in the lower panel. *Bam*HI and *Eco*RI restriction sites of the pGEX6P-1-encoded Gly-Ser (GS) and Glu-Phe (EF), respectively (indicated with underlines).

We generated mAbs against GST by immunizing mice with GST protein bacterially expressed using the pGEX-2T vector, resulting in the establishment of several hybridoma clones. During characterization of the mAbs by Western blotting, we noticed that mAb G196 recognized the proteins encoded by pGEX-4T-2 (4T-2, Fig. 1a) and by pGEX-2T (data not shown), but not the proteins encoded by pGEX-6P-1 (6P-1, Fig. 1a) or by pGEX-3X (data not shown). As expected, mAb G196 failed to detect the core domain of GST protein (2–212) (core, Fig. 1), indicating that the G196 epitope falls within the overlapping C-terminal extension of the proteins encoded by pGEX-2T and pGEX-4T-2 (Fig. 1a, lower panel).

mAb G196 also recognized the GST fusion proteins encoded by pGEX-2T or pGEX-4T-2, in which cDNAs were inserted using *Bam*HI and *Eco*RI restriction sites (data not shown). This suggested that the G196 epitope extends from the end of the GST core to Gly-Ser (encoded by the *Bam*HI site) (underlined GS, Fig. 1a, lower panel).

### Precise determination of the G196 mAb epitope

To confirm that the G196 epitope is located on the C-terminal extension of the 4T-2 fragment, and to further determine the precise location of the G196 mAb epitope, double-stranded oligonucleotides encoding peptides containing the C-terminal extension were inserted into the *Bam*HI/*Eco*RI restriction sites within the pGEX-6P-1 vector. Total protein lysates were prepared from *Escherichia coli* that had been transformed with individual GST fusion protein constructs. The lysates were separated using two SDS-PAGE gels: one gel was stained with Coomassie Brilliant Blue to determine the equivalency of expression and loading, and the other (1/10 sample volume applied) was examined by Western blotting with mAb G196. mAb G196 detected 6P-11, 6P-12, 6P-13, 6P-14, 6P-15, and 4T-2 as a positive control (Fig. 1b and c), whereas mAb G196 did not react with 6P-16, 6P-17, 6P-19, or 6P-1 as a negative control (Fig. 1c). These results identified the minimal epitope as the five amino acid sequence DLVPR.

**Figure 2:**
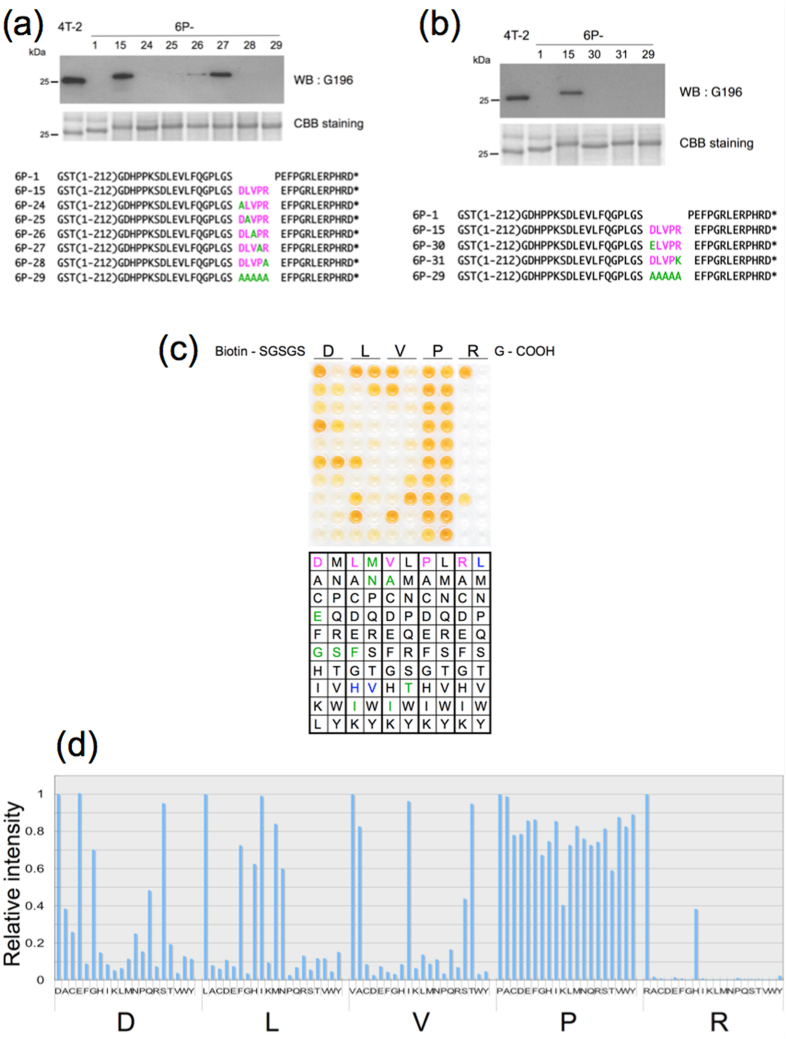
Refinement of mAb G196 epitope. (**a**) and (**b**) Western blot analysis using mAb G196 (upper panel) and Coomassie Brilliant blue staining (middle panel) of the bacterially expressed proteins shown in the lower panel. *Bam*HI and *Eco*RI restriction sites of the pGEX6P-1-encoded Gly-Ser (GS) and Glu-Phe (EF), respectively (indicated with underlines). (**c**) A representative permutation ELISA analysis of the G196 epitope. N-terminal biotin coupled 11-aa peptides encompassing the G196 epitope SGSGSDLVPRG were permutated at single positions (underlined) to the 19 remaining coded amino acids. The peptides were immobilized onto streptavidin-coated plates, incubated with mAb G196, washed, and then the bound antibody was detected using an HRP-labeled anti-mouse antibody. (**d**) Densitometric analysis of the relative intensities of duplicate-mean spots from the permutation analysis.

We conducted alanine scanning mutagenesis on the epitope to determine which amino acid residues were responsible for mAb recognition. mAb G196 detected 6P-27 (Pro to Ala at position 4). In contrast, G196 only faintly detected 6P-26 (Val to Ala at position 3) and did not detect 6P-24, 6P-25, 6P-28, or 6P-29 (Fig. 2a). These results clarified that the epitope contains four critical residues and one nonessential residue (Pro at position 4) under denaturing conditions.

The G196 epitope harbors a negatively charged amino acid (Asp at position 1) and a positively charged amino acid (Arg at position 5) at opposite ends of the sequence. To investigate the contributions of the charged amino acid residues of the epitope to mAb binding, we substituted both residues with physicochemically similar amino acids (6P-30: Asp to Glu at position 1; 6P-31: Arg to Lys at position 5). Replacement of either residue did not salvage immunoreactivity (6P-30 or 6P-31, Fig. 2b). Western blot analysis revealed that these charged amino acids at opposite ends of the epitope are critical residues for G196 antibody binding under denaturing conditions.

To evaluate the importance of each amino acid residue of the DLVPR epitope for mAb recognition under non-denaturing conditions, we conducted permutation analysis using peptides coupled through their N-termini to biotin. Single positions of the underlined residues in the peptide SGSGSDLVPRG were substituted individually with the other 19 coded amino acids. The peptides were immobilized onto streptavidin-coated plates, incubated with mAb G196, washed, and then the bound antibody was detected using an HRP-labeled anti-mouse secondary antibody (Fig. 2c,d). The results showed that Asp at position 1 can be replaced by Glu and the flexible amino acids Gly and Ser, whereas Leu at position 2 can be changed to one of several hydrophobic amino acids (Ile, Met, Phe, Asn) but not to Val, and to the hydrophilic amino acid His. Val at position 3 can be exchanged with one of two hydrophobic amino acids (Ile, Ala), and also with the hydrophilic amino acid Thr. Pro at position 4 does not show interaction specificity, whereas Arg at the last position is the most specific: Arg can not be substituted with Lys.

### Binding thermodynamics of G196 antigen binding fragment to the epitope peptide

**Figure 3:**
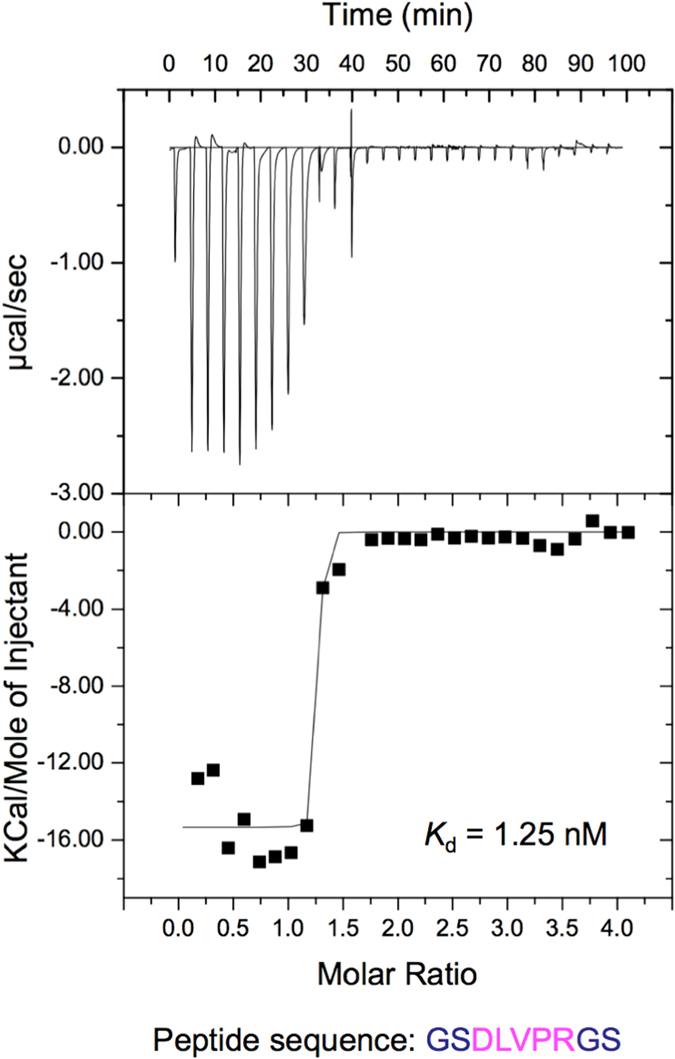
Isothermal titration calorimetry binding profile of G196 IgG Fab with the representative synthetic peptide GSDLVPRGS. The *top panel* shows a typical calorimetric titration of 25 μM G196 IgG Fab with synthetic peptide at 25 °C. The *bottom panel* shows the integrated curve showing the experimentally obtained (◾) points and the best fit (−). The best fit to the data yielded *n* = 1.2 sites, *K*_d_ = 1.25 nM (±0.77), Δ*H* = −15.35 kcal/mol, and T*ΔS* = −3.19 kcal/mol.

We used isothermal titration calorimetry (ITC) to investigate the thermodynamic binding properties of G196 antigen binding fragment (Fab) against a representative epitope peptide (GS**DLVPR**GS). A substantial exothermic reaction (change in enthalpy Δ*H* = −15.35 kcal mol^−1^) was observed upon mixing, resulting from a high-affinity binding event (dissociation constant *K*_d_ = 1.25 ± 0.77 nM) with 1:1 stoichiometry (*n* = 1.2 ±  0.05) (Fig. 3).

### Crystal Structure of G196 Fab

**Figure 4:**
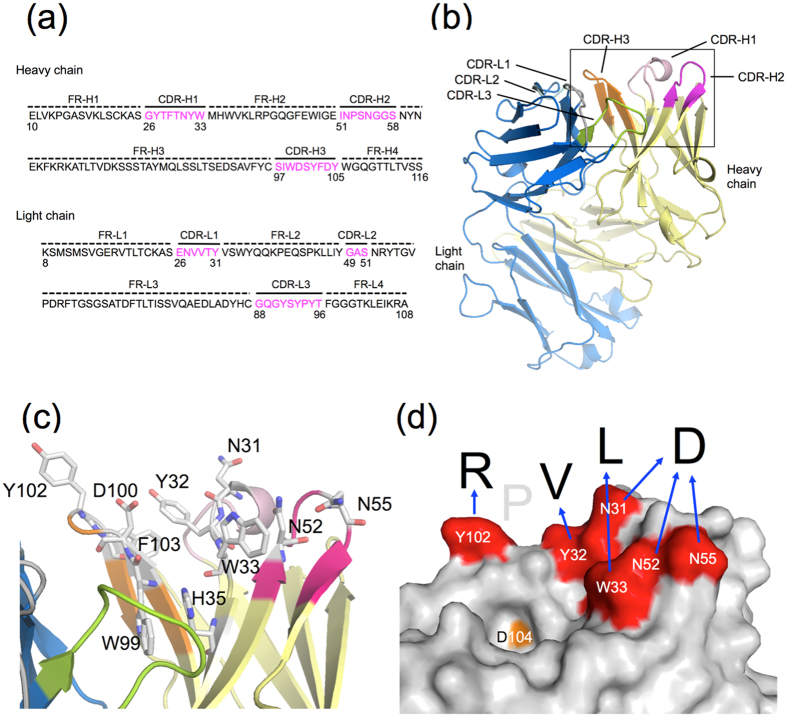
Structure of G196 IgG Fab. (**a**) Amino acid sequences of the heavy and light chain variable regions of mAb G196. (**b**) Ribbon representation of the Fab fragment of mAb G196. CDR-H1, CDR-H2, CDR-H3, other heavy chain, CDR-L1, CDR-L2, CDR-L3, and the other light chain are colored *pink, hotpink, orange, yellow, gray, palecyan, green* and *blue*, respectively. The region surrounded by a square is enlarged in (**c**). (**d**) Speculative model for the recognition of mAb G196 with the G196 epitope DLVPR. Putative recognition amino acids against DLVPR and Asp104 are colored *red* and orange, respectively.

The structure of G196 Fab was determined as the antigen-free form at 2.0 Å resolution using the molecular replacement method. Data and refinement statistics are summarized in Table 1. The *R* and *R*_free_ values of the final model of G196 Fab were 0.191 and 0.228, respectively. The Fab fragment displays a conventional immunoglobulin fold characterized by an anti-parallel β-sheet sandwich architecture, with an r.m.s.d. value of 1.76 Å to anti-phenobarbital mAb Fab structure (Protein Data Bank code 1IGY). Trp99 in complementarity-determining region (CDR)-H3 interacts with His35 in framework region (FR)-H2 through π-π aromatic -stacking and forms hydrophobic core with Tyr32 and Trp33 in CDR-H1, and Phe103 in CDR-H3 (Fig. 4). Asn-31 in CDR-H1, Asn52 and Asn55 in CDR-H2, are located at one side of the hydrophobic core.

### G196-tag application for scientific research

**Figure 5:**
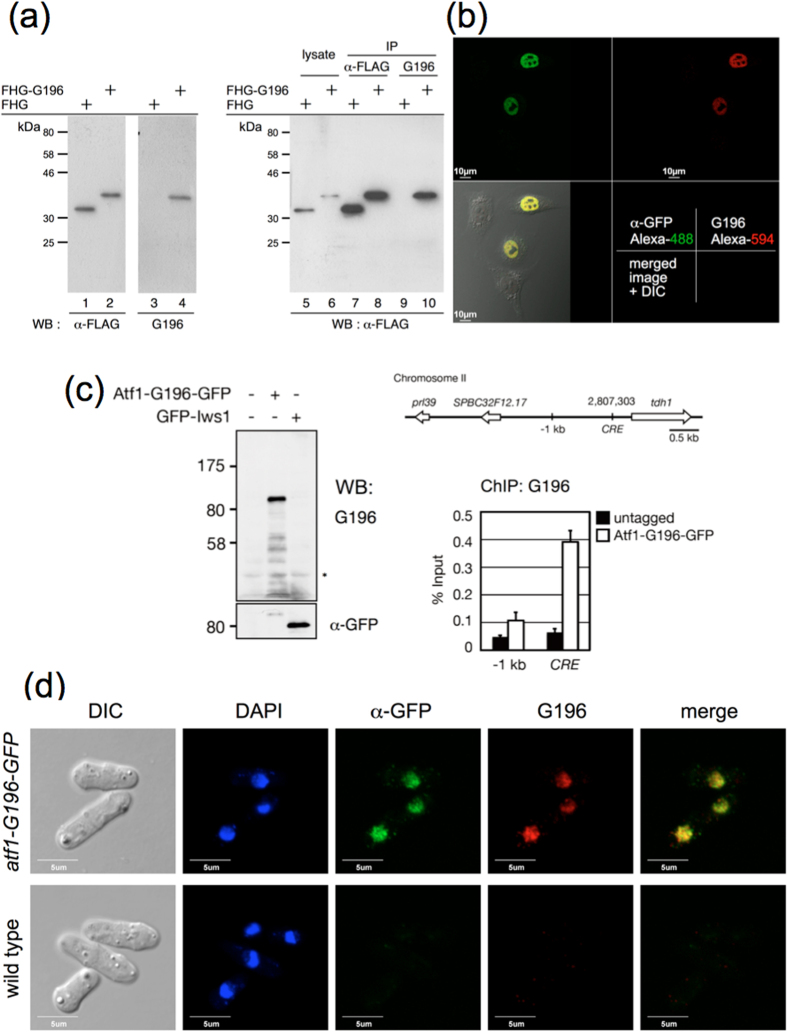
Application of the G196 epitope tag system for scientific research. (**a**) Western blot and immunoprecipitation (IP) analysis of FLAG-HA-Emerald GFP (FHG) tagged with C-terminal G196-tag in HeLa cells using mAb G196. HeLa cells were transfected with FHG/pcDNA3 or FHG-G196/pcDNA3. After incubation for two days, the cells were lysed and subjected to Western blotting with anti-FLAG (M2) or G196 mAbs (*left panel*). These lysates were immunoprecipitated with anti-FLAG (M2) (lanes 7, 8) or G196 (lanes 9, 10) mAbs and subjected to Western blotting with anti-FLAG polyclonal antibody (*right panel*). (**b**) Immunofluorescence of GFP- and G196-tagged nuclear protein in HeLa cells using mAb G196. HeLa cells were transfected with FHG-G196-NAC1/pcDNA3. After incubation for two days, the cells were fixed and incubated with mAb G196 and anti-GFP polyclonal Ab, then stained with Alexa-594 anti-mouse and Alexa-488 anti-rabbit secondary antibodies. (**c**) Western blot and chromatin immunoprecipitation (ChIP) analysis of Atf1-G196-GFP in fission yeast using mAb G196. A DNA fragment containing the transcription factor *atf1* gene fused with the *G196* epitope-*GFP* gene was replaced with the genomic *atf1* gene. Protein extracts were separated by SDS-PAGE and detected with mAb G196 and anti-GFP polyclonal antibody (*left panel*). GFP-Iws1 expressing cells were used as a negative control. Asterisk indicates non-specific band also detected using the second Ab alone. Schematic representation of the *tdh1* locus (*right upper panel*). The promoter of the *tdh1* gene harbors a CRE consensus site. ChIP-qPCR analysis of the Arf1-G196-GFP level in the indicated regions (*right lower panel*). (**d**) Immunofluorescence of GFP- and G196-tagged nuclear protein in fission yeast cells using mAb G196. Atf1-G196-GFP expressing cells were fixed and incubated with mAb G196 and anti-GFP polyclonal Ab, then stained with Alexa-594 anti-mouse and Alexa-488 anti-rabbit secondary antibodies.

Short peptides and paired mAbs specific for the sequences of interest are powerful and irreplaceable tools for scientific research. To ascertain the possible versatility of G196-tag as a versatile fusion tag, we generated several human and yeast expression constructs in which the G196-tag was fused to reporter proteins. First, we expressed FLAG- and HA-tagged Emerald GFP (hereafter abbreviated FHG) as a reporter protein, C-terminally fused with a G196-tag (6P12, SDLVPRGSP) in HeLa cells. As shown in Fig. 5a, reporter protein fused with the G196-tag (FHG-G196) was detected as a single band in cell extracts by Western blotting with both anti-FLAG and the G196 mAbs (lanes 2, 4), whereas, as expected, the reporter without G196-tag (FHG) was not detected by mAb G196 (lane 3). It is noteworthy that mAb G196 did not cross-react with cellular proteins, and reacted solely with the G196-tagged protein (lanes 3, 4). In addition, mAb G196 efficiently immunoprecipited reporter protein fused with the G196-tag (lane 10) from cell extracts, comparable to that of FLAG immunoprecipitates (lane 8), but did not immunoprecipitate reporter protein lacking the G196-tag (lane 9).

Second, we applied the G196 epitope tag system to immunofluorescence assays with HeLa cells expressing the G196-tagged reporter protein to evaluate its utility. We utilized the cancer-related transcription regulator protein NAC1 as a nuclear reporter protein^18^,^19^. mAb G196 successfully detected the nuclear reporter protein, as did the control polyclonal anti-GFP antibody (Fig. 5b).

Lastly, we applied the G196 epitope tag system to yeast cells. The Atf1/Pcr1 heterodimeric transcription factor binds the cyclic AMP-responsive element (CRE)-like hexanucleotide sequence 5′-TGACGT-3′^20^. We expressed C-terminally 3 × G196-tagged Atf1 as a reporter protein in fission yeast. Western blotting showed that mAb G196 detected the Atf1 protein in yeast cell extracts, and chromatin immunoprecipitation (ChIP) enriched the Atf1 protein at the promoter of the *tdh1* gene, which harbors a CRE consensus site^21^ (Fig. 5c). Furthermore, mAb G196 recognized G196- and GFP-tagged Atf1, as shown by immunofluorescent staining of yeast, comparable to that of a polyclonal anti-GFP antibody (Fig. 5d). These results indicate that the G196 epitope tag system is suitable for Western blotting, immunoprecipitation, ChIP, and immunofluorescence assay in both yeast and human.

## Discussion

Here we describe the characterization of a new G196 epitope tag system exhibiting defined properties. The G196 epitope tag system was characterized using Western blotting, immunofluorescence, and immunoprecipitation using human cells (this study and ref. 22) and Western blotting, immunofluorescence, immunoprecipitation, and chromatin immunoprecipitation using yeast cells (this study and refs 23 and 24). The new G196 epitope tag system will thus be useful for a broad range of studies in cell biology and biochemistry.

The minimal epitope of the G196 mAb is the five amino acid sequence DLVPR. We typically add a glycine-serine linker sequence upstream and downstream of the minimal epitope to minimize the influence of the tag on the target protein and maximize its accessibility for antibody binding, and therefore we used the nine amino acid sequence GSDLVPRGS as the original G196-tag. mAb G196 detected both N- and C-terminally G196-tagged protein (this study and refs. 22 and 23). The Grand Average of Hydropathicity score of the peptide was −0.444 (http://www.bioinformatics.org/sms2/protein_gravy.html), indicating that the peptide was hydrophilic; indeed, 1 mg of the synthetic peptide was dissolved in 1 mL of PBS and used for competitive elution of bound G196-tagged proteins from a G196 affinity column (data not shown).

A *K*_d_ value of ~10 nM or less between the anti-tag mAb and the tag is desirable for rapid and robust purification of modest to low abundance proteins of interest, and such levels are common when the proteins are expressed at the endogenous level^25^. It is noteworthy that the *K*_d_ between mAb G196 and the G196-tag peptide is 1.25 nM (Fig. 3), which is comparable to or higher than the affinity of the anti-FLAG M2 mAb/FLAG-tag interaction (*K*_d_ = 3–28 nM, and typically considered high affinity)^26^,^27^,^28^. It was previously reported that the *K*_d_ of the anti-c-Myc 9E10 mAb/ c-Myc-tag is 2.2–560 nM^28^,^29^,^30^. Although the affinity of the anti-FLAG M2 mAb/FLAG-tag interaction exhibited a low-nM *K*_d_ as described above, the 3 × tag version was superior in Western blotting, with a sensitivity reportedly increased by over an order of magnitude^7^,^31^. In our hands, the 3 × G196-tag version exhibited higher affinity compared to G196-tag in yeast (this study and refs 23 and 24).

Western blotting is widely used to ascertain an antibody’s specificity and is an appropriate first validation step. mAb G196 detected the G196-tagged reporter protein as a single band at the proper molecular weight by Western blotting in HeLa cells, a human cell line (Fig. 5a, lane 4). Given that decreasing the length of a peptide tag has the disadvantage of increasing its reactivity with endogenous proteins containing the same sequence, we were surprised to observe that mAb G196 did not (cross-)react with cellular proteins, and only reacted with G196-tagged protein in HeLa cells (Fig. 5a, lanes 3) and several other human cell lines (data not shown). Use of the G196 epitope DLVPR as a search sequence for scanning the human proteome, disregarding splice variants, with ScanProsite^32^ results in 11 hits on all UniProtKB/Swiss-Prot database sequences (release 2016_08 of 07-Sep-16: 551987 entries) (Supplementary Table 1). It is probable that the transfected G196-tagged reporter protein was overexpressed at a higher level than these 11 proteins were endogenously expressed. Alternatively, these 11 proteins were weakly expressed or did not express in these cell lines. mAb G196 must be used with caution when G196-tagged proteins are immunoprecipitated in order to search for binding partners in human cells, or when G196-tagged proteins are being investigated in cells for the first time. Cross-reactions of specific cellular proteins with anti-tag mAbs are observed universally. For example, anti-FLAG M2 mAb showed non-specific Western blot bands in Indian mustard plant^33^, tobacco^34^ and rat brain tissue^35^. ScanProsite is an informative tool for checking (cross-)reactions of specific cellular proteins with anti-tag mAbs, and is available free of charge at http://prosite.expasy.org/scanprosite/.

Although not described in detail in the literature, many researchers have noticed that anti-FLAG antibody M2 is inadequate for the immunofluorescence staining of cells to detect FLAG-tagged proteins expressed in the cell nucleus. Previously, we studied the subcellular localization of myocardin-related transcription factor (MRTF)-B, a shuttle protein between the nucleus and cytoplasm that is expressed in response to various stimuli. FLAG-tagged MRTF-B in the nuclei of A431 cells was detectable, but stained more faintly compared to FLAG-tagged MRTF-B in the cytoplasm reacted with anti-FLAG mAb M2. We then changed the epitope tag system from FLAG-tag to G196-tag and could detect G196-tagged MRTF-B in the nuclei as well as in the cytoplasm, thus allowing evaluation of the relative subcellular distribution of MRTF-B^22^. In this study, we clearly stained G196-tagged NAC1 and ATF1 in the nuclei of human and yeast cells, respectively (Fig. 5b,d), thus demonstrating the utility of the G196 epitope tag system for the immunofluorescence staining of cells to detect G196-tagged proteins expressed in the cell nucleus. It has been reported that Arg at mild pH (pH 3.5–4.4) can effectively dissociate FLAG-tagged proteins from an anti-FLAG M2 mAb affinity column for use in fusion protein purification technology^36^. Arg and Lys are abundant (comprising more than 20% of the amino acids) in histone proteins^37^ and histones proteins have been estimated to represent as much as 55% of the nuclear proteins^38^. The high abundance of Arg and Lys in the cell nucleus might interfere with anti-FLAG mAb M2 recognizing FLAG-tagged nuclear proteins. Recently, a novel anti-FLAG mAb (2H8, mouse IgG_2a_) with a sensitivity higher than that of M2 was generated and shown to be suitable for immunofluorescence and immunocytochemistry studies^39^, but mAb 2H8 can recognize only amino-terminal FLAG tags.

Although the structure of G196 Fab shown in this study does not contain the epitope peptide, it nonetheless shows remarkable features of the antigen binding site (Fig. 4). Tyr32 and Trp33 in CDR-H1, and Trp99 and Phe103 in CDR-H3, together form a hydrophobic core. Leu at position 2 and Val at position 3 of the G196 epitope might be recognized in the region. In particular, Leu can bind through stacking interaction with aromatic residues, given that it can be replaced with His or Phe (Fig. 2c,d). Arg at position 5 might interact with an acidic residue, such as Asp104 in CDR-H3. However, Asp104 is too close to the hydrophobic region for this interaction with Arg to occur, as demonstrated by the finding that the residue at position 4 in the epitope can be replaced with any other residue (Fig. 2c). Also, Arg can be replaced with His but not with Lys. Therefore, it is likely that Arg at position 5 is recognized by Tyr102 in CDR-H3 rather than by Asp104. On the other hand, the Asp residue at position 1 of the epitope can be replaced not only with Glu but also Ser and Gln. Asn31 in CDR-H1, and Asn52 and Asn55 in CDR-H2, are located at the side of the hydrophobic core and are good candidates for recognizing Asp. Structural analysis of G196 complexed with the epitope peptide will allow precise elucidation of this antibody’s high affinity recognition mechanism for the epitope.

Each epitope tag system, including G196 epitope tag system, has its own distinct advantages and drawbacks, and it is important to consider these advantages and drawbacks prior to final selection of the tag to be used. In the future, we intend to apply the G196 epitope tag system 1) as a bispecific antibody for drug targeting and 2) to structural studies of membrane proteins, including G-protein-coupled receptors.

## Materials and Methods

### Antibodies

The following commercial antibodies were used: mouse monoclonal anti-FLAG (M2, Sigma-Aldrich, St. Louis, MO, USA); rabbit polyclonal anti-FLAG (60–031, BioAcademia, Osaka, Japan) and anti-GFP (60–011, BioAcademia); HRP-conjugated goat F(ab’)_2_ anti-mouse (710–1332, Rockland Immunochemicals, Limerick, ME, USA) and goat anti-rabbit IgG (111–035–003, Jackson ImmunoResearch Laboratories, West Grove, PA, USA); Alexa 488-conjugated goat anti-rabbit IgG(H+L) (A-11034, ThermoFisher Scientific, Waltham, MA, USA) and Alexa 594-conjugated goat anti-mouse IgG(H+L) (A-11032, ThermoFisher Scientific).

### Plasmid construction

All short 4T-2 fragments were generated by annealing complementary oligos. The oligos used to synthesize the indicated fragments are summarized (Fasmac, Kanagawa, Japan) in Supplementary Table 2. Annealing reaction mixtures contained equimolar amounts of each oligo and the reactions were performed in a thermal cycler, with initial denaturation at 98 °C for 3 min, followed by a temperature decrease of 2 °C every 20 s down to 4 °C to allow the oligos to anneal slowly. The oligos contained the *Bam*HI/*Eco*RI restriction sites and the 4T-2 fragments were cloned into these restriction sites within the pGEX-6P-1 vector (GE Healthcare, Buckinghamshire, England).

A fragment of the GST gene, encoding residues 2–212, was prepared by polymerase chain reaction (PCR) amplification using the Pfu polymerase (BioAcademia) and the pGEX-6P-1 plasmid as the template. The primers contained the *Bam*HI and *Xho*I restriction sites (underlined) and their sequences were 5′-GGGGATCCTCCCCTATACTAGGTTAT-3′ (GST-6P22-S) and 5′-GGCTCGAGTTAACCACCAAACGTGGCTTG-3′ (GST-6P21-AS). The amplified fragments were digested with *Bam*HI and *Xho*I, then cloned into the pET28a vector (Merck Millipore, Darmstadt, Germany) digested with the same two enzymes. BL21 (DE3) *E. coli* were transformed with the plasmids, and protein expression was induced with 0.1 mM isopropyl-β-D-1-thiogalactopyranoside (IPTG). The final constructs were sequenced to ensure that no mutations had occurred during the PCR and cloning processes. The expressed proteins were purified as described previously^40^.

### mAb generation

Mouse mAb G196 was generated by immunizing mice with GST protein bacterially expressed using the pGEX-2T vector, and serum titers were monitored by immunoblotting using the same GST protein. Clonal populations of fusion cells were screened by ELISA for antibody production against this GST protein. Productive cells were cloned to monoclonal lines by serial dilution screening. Highly concentrated mAbs were isolated from murine ascites after an intraperitoneal injection of hybridoma cells. All animal experiments were performed in compliance with the standards established by the International Guiding Principles for Biomedical Research Involving Animals and were approved by the animal study committee of Shimane University.

### Cell culture and transfection

The HeLa human cervical epithelioid carcinoma cell line was purchased from the Japanese Collection of Research Bioresources (JCRB) Cell Bank (JCRB9004). HeLa cells and hybridomas were grown in DMEM (Nissui, Tokyo, Japan) and RPMI1640 medium, respectively, supplemented with 10% FBS (Sigma-Aldrich). HeLa cells were transiently transfected with each plasmid using Lipofectamine 2000 (ThermoFisher Scientific) according to the manufacturer’s instructions.

### Permutation ELISA analysis

The biotinylated 11-aa peptide (biotin-SGSGSDLVPRG) was synthesized by Mimotopes (Clayton, VIC, Australia). The sequence of the G196 epitope was DLVPR and the 5-aa oligopeptide of SGSGS was used as a spacer between biotin and the G196 epitope. Single positions of the peptide biotin-SGSGSDLVPRG (epitope sequence underlined) were substituted with the other 19 coded amino acids. The biotinylated peptides had >70% purity as judged by high-performance liquid chromatography.

Permutation ELISA analysis was performed according to the manufacturer’s instruction. Briefly, the biotinylated peptide was applied to NeutrAvidin-coated wells of a 96-well plate (15125, ThermoFisher Scientific) (5 μg/mL, 1 h, 37 °C). The plate was blocked (2% BSA in PBS, 1 h, 22 °C), then washed 4 times with PBST (PBS buffer containing 0.1% Tween 20). Subsequently, 100 μL of a 1:2,000 dilution of purified mAb G196 (1 mg/mL) was added to the wells for 1 h at 22 °C. After washing 4 times with PBST, 100 μL of HRP-conjugated goat F(ab’)_2_ anti-mouse antibody (1:5,000 diluted with 2% BSA/PBS) was added and the plate was incubated at 22 °C for 1 h. After washing 4 times with PBST and twice with PBS, 100 μL of *o*-phenylenediamine dihydrochloride peroxidase substrate (P1526, Sigma-Aldrich) was added and the plate was incubated at 22 °C for 15 min. The reaction was stopped by the addition of 100 μL of 2 M H_2_SO_4_, and the OD value at 492 nm was measured using a microplate reader.

### Isothermal Titration Calorimetry (ITC)

ITC was performed with a VP-ITC titration calorimeter (MicroCal, GE Healthcare, Buckinghamshire, England) at 25 °C. Calorimetric measurements were carried out with purified G196 Fab and the synthetic peptide GSDLVPRGS (Tufts University Core Facility, Boston, MA, USA). The ITC cell (1.4 mL) contained 25 μM antibody G196 Fab in 20 mM Tris-HCl (pH 8.0), 150 mM NaCl, and was stirred constantly at 307 rpm throughout the experiment. The peptide (500 μM) in the same buffer was added using 28 injections: the first injection volume was 3 μL and all subsequent injections were 10 μL. The first data point was not used for fitting. Binding parameters were determined using the Origin software package provided with the instrument by fitting the data to a single-site model.

### Sequencing of the variable region of the mAb G196

Total RNA was extracted from 3 × 10^6^ cells of the murine hybridoma clone G196–3 by using Sepasol-RNA I Super G (Nacalai Tesque, Kyoto, Japan). Total RNA was reverse transcribed into cDNA by using oligo-dT_14_ and ReverTra Ace (mutated M-MLV reverse transcriptase, RNaseH negative) (TOYOBO, Osaka, Japan). DNA fragments encoding the heavy and light chain were PCR amplified using the Pfu polymerase (BioAcademia). The primers used, containing the *Bam*HI and *Eco*RI restriction sites, for amplifying heavy chain genes were VH1-1S and IgG2-1AS; for light chain genes the primers were VK-1S and CK-2AS (Supplementary Table 2). The amplified fragments were digested with *Bam*HI and *Eco*RI and cloned into the pBlueScript II SK(+) vector (Agilent Technologies, Santa Clara, CA, USA) digested with the same two enzymes. The DNA sequences of the heavy and light chain genes were determined by automated sequencing using T7 and T3 sequencing primers with BigDye sequencing reagent (ThermoFisher Scientific) and an ABI 3100 automated capillary DNA sequencer (ThermoFisher Scientific).

The isotype of mAb G196 was identified as mouse IgG_1_ using an IsoStrip mouse monoclonal antibody isotyping kit (Roche Diagnostics, Basel, Switzerland).

### Purification of Fab fragments from mAb G196

mAb G196 IgG was purified from ascites of hybridoma-bearing mice as follows. The ascites were diluted 1:40 with 20 mM Tris-HCl (pH 8.0), 150 mM NaCl, and were purified by passage though four chromatography columns (HiTrap Q HP, GE Healthcare; hydroxyapatite, Bio-Rad Laboratories, Hercules, CA, USA; HiTrap SP HP, GE Healthcare; HiTrap Heparin HP, GE Healthcare). The flow-through was precipitated using ammonium sulfate. The resulting precipitate was suspended in 1/100 of the starting volume with 20 mM sodium phosphate (pH 7.0), and was dialyzed against 20 mM Tris-HCl (pH 8.0). The material was further fractionated by FPLC on a HiTrap Q HP column (GE Healthcare). Proteins were eluted using a stepwise NaCl gradient, with the final concentration being 150 mM NaCl. Purified antibody was fragmented using papain, and the resulting Fab fragments were purified by protein A chromatography (Pierce Fab preparation kit, ThermoFisher Scientific). The purity and homogeneity of the fragment were evaluated by means of SDS-PAGE followed by Coomassie Brilliant Blue staining.

### Crystallization and Structure Determination

mAb G196 Fab was crystallized using the hanging drop vapor diffusion method by mixing 1 μL sample solution (8 mg/mL) and 1 μL reservoir solution (100 mM sodium citrate (pH 5.0), 20% PEG 20000) equilibrated against 1 mL reservoir solution. The crystals grew at 20 °C in 15% PEG 20000, 100 mM sodium citrate (pH 5.0) over a period of one week. The crystals were briefly soaked in a cryoprotectant solution consisting of the reservoir solution plus 20% (v/v) glycerol before cryocooling. X-ray data were collected using an ADSC Q270 CCD detector at beam-line BL17A of the Photon Factory, Tsukuba, Japan. The wavelength of the incident X-rays was 0.98 Å. Diffraction data sets were processed with HKL2000^41^ and scaled with SCALEPACK^41^. The crystals belonged to the space group *P*212121, with unit cell parameters of *a* = 38.65 Å, *b* = 82.27 Å, *c* = 127.31 Å. The structures were solved by molecular replacement using PHASER^42^ and the previously reported structures of the heavy and light chain of anti-(4-hydroxy-3-nitrophenyl)acetate and anti-phenobarbital mAbs (PDB codes 1NGQ and 1IGY, respectively) as starting models. COOT^43^ and REFMAC5^44^ were subsequently employed for iterative cycles of model building and refinement. The later stages of crystallographic refinement were carried out using PHENIX^45^. After an initial round of simulated annealing refinement, several macrocycles that included bulk solvent correction, anisotropic scaling of the data, individual coordinate refinement with minimization, and individual isotropic ADP (atomic displacement parameters) refinement, were carried out with maximum likelihood as the target function. In the course of the refinement, water molecules were added to the models by manual inspection of their positions in both the 2*F*o – *F*c and *F*o – *F*c maps, and combined TLS (translation, libration, and screw-rotation) and individual ADP refinement were carried out in the final stages. Manipulation of the model and validation were performed with COOT^43^. The stereochemistry of the final model was also assessed using PROCHECK^46^. Data collection and refinement statistics are summarized in Table 1.

The atomic coordinates and experimental structure factors of mAb G196 Fab have been deposited with the Protein Data Bank under code 5H2B.

### Yeast cell manipulation

The fission yeast strains used are listed in Supplementary Table 3. Yeast extract medium (YES) was supplemented with 250 mg/L each of adenine, leucine and uracil. Atf1 and Iws1 were tagged by modifying the 2-step PCR method to create a fragment for gene targeting. The primers used for genetic manipulations are listed in Supplementary Table 2.

C-terminal tagging of the *atf1* gene (Supplementary Fig. 1) was accomplished using the G196t2 primer. This primer contains a sequence homologous to a portion of the *atf1* coding sequence, a sequence for 3 × G196-tag, and a sequence homologous to another primer, com5. In the first PCR step, two sequences flanking the translational stop codon of the *atf1* gene were amplified with the primers t1 and G196t2, and with the primers t3 and t4, using the genomic DNA as a template. The GFP-kanMX cassette was also amplified with the primers com5 and com6, using pFA6a-GFP(S65T)-kanMX as a template. The three PCR products were fused together in the second PCR step with the primers t1 and t4 to produce a DNA fragment for homologous recombination.

N-terminal tagging of the *iws1* gene (Supplementary Fig. 2) was accomplished by amplifying the kanMX cassette from pFA6a-kanMX with the primers Nt3 and Nt4 in the first PCR step. The EmGFP coding sequence of EmGFP/pCDNA3.1-neo was amplified with the primers Nt7 and Nt8. We also amplified three sequences from the *iws1* locus: one homologous to the upstream region of the *iws1* promoter (upstream), one homologous to the promoter (promoter), and one homologous to a portion of the *iws1* coding region (CDS). The three sequences were amplified as follows: upstream sequence using the primers Nt1 and Nt2, the promoter sequence using Nt5 and NT6, and the CDS sequence using Nt9 and Nt10. The Nt2, Nt5, Nt6 and Nt9 sequences contained a sequence homologous to Nt3, Nt4, Nt7 and Nt8, respectively. The five fragments produced in the first PCR step were fused together in the second PCR step with the primers Nt1 and Nt10 to produce a DNA fragment for homologous recombination.

Yeast transformation was conducted using a yeast transformation kit for *Saccharomyces pombe* (WAKO Pure Chemical Industries, Osaka, Japan) according to the manufacturer’s instructions. G418 sulfate (Merck) was added to YES at 100 mg/L to select the recombinants. The genomic DNA of the strains was Sanger-sequenced to confirm that proper recombination had occurred and that no additional mutation had been introduced during the procedure.

Cells (1 × 10^8^) growing exponentially in YES medium at 30 °C were harvested to obtain whole cell extracts as described elsewhere^47^. Samples were resolved by SDS-PAGE on 7% gels, semi-dry blotted to PVDF membranes, and detected using an ECL detection system (PerkinElmer, Waltham, MA, USA). Mouse mAb G196 (1:2000) and anti-GFP rabbit polyclonal antibody (1:1000) were used as primary antibodies. HRP-conjugated goat anti-mouse antibody (1:5000, Rockland Immunochemicals) and anti-rabbit antibody (1:2000, Jackson ImmunoResearch Laboratories) were used as secondary antibodies.

### Chromatin immunoprecipitation

Yeast cells were grown in YES medium at 30 °C to mid-log phase. After formaldehyde crosslinking and quenching, 1 × 10^8^ cells were harvested to obtain 2 mL of soluble input extract. M-280 anti-mouse sheep antibody-conjugated magnetic beads (112-02, ThermoFisher Scientific) were used for immunoprecipitation: 200 μL of the beads were washed twice with extraction buffer (50 mM HEPES-KOH (pH 7.5), 140 mM NaCl, 1 mM EDTA, 1% Triton X-100, and 0.1% sodium deoxycholate), and then incubated in 1 mL of extraction buffer containing 3 μL of mAb G196 for 1 h at 4 °C. The beads were again washed twice with extraction buffer to remove unbound antibodies and incubated for 1 h with 800 μL of input extract at 4 °C. The relative concentrations of the target sequences were determined using SYBR Premix ExTaq (RR041A, TaKaRa Bio, Shiga, Japan) and a Thermal Cycler Dice Real Time System TP800 (TaKaRa Bio), according to the manufacturer’s instructions. The primers for detecting the −1 kb region were 5′-GAATCGATGCTCATTTGAGTC-3′ and 5′-TACTCCACCATAGTTCTTGTC-3′, and for the *tdh1 CRE* region the primers were 5′-GTGCTAGCAATTCCTCCTTC-3′ and 5′-TTCGTTGGTAATCTGCCTCG-3′.

### Immunofluorescence microscopy

HeLa cells were simultaneously fixed and permeabilized for 10 min with 3.7% formaldehyde and 0.2% Triton X-100 in PBS, washed 3 times with PBS, and then blocked for 30 min with 5% skim milk in PBS. Next, the cells were incubated with primary antibodies for 1 h at room temperature. After four washes with PBS, secondary antibody incubations were performed on coverslips for 1 h at room temperature, then the coverslips were mounted (mounting medium; Vector Laboratories, Burlingame, CA, USA) with DAPI.

Yeast cells exponentially growing in YES medium at 30 °C were fixed with 3% formaldehyde and incubated at 18 °C for 30 min. After quenching with 250 mM glycine, the cells were washed with PBSEMS (PBS buffer with 1 mM EGTA, 1 mM MgSO_4_ and 1.2 M sorbitol), resuspended in PBSEMS containing 5 mg/mL Zymolyase 20T (07663-91, Nacalai Tesque) and 0.1% 2-mercaptoethanol, and incubated at 37 °C for 1 h. The cells were washed three times with PBSEMS and resuspended in PBSEMT (PBS with 1 mM EGTA, 1 mM MgSO_4_ and 1% Triton X-100) and incubated at room temperature for 30 sec. After washing three times with PBSEM (PBS with 1 mM EGTA and 1 mM MgSO_4_), the cells were incubated in PBSEMALM (PBS with 1 mM EGTA, 1 mM MgSO_4_, 0.1% sodium azide, 0.1 M L-lysine and 5% skim milk) for 30 min. The cells were resuspended in PBSEMALM containing G196 mAb (1:1000) and anti-GFP rabbit polyclonal antibody (1:1000). After overnight incubation at room temperature, cells were washed with PBSEMALM three times, then resuspended in PBSEMALM containing anti-mouse antibody conjugated with Alexa 594 (1:100) and anti-rabbit conjugated with Alexa 488 (1:100). After one-hour incubation, the cells were washed with PBSEMAL containing 5% skim milk three times and resuspended in PBS containing 0.1% sodium azide. The cell suspension was mixed with an equal amount of 1% low-melting point agarose with DAPI on glass plates.

Cells were observed under a confocal microscope (FV1000, Olympus, Tokyo, Japan).

## Additional Information

**How to cite this article**: Tatsumi, K. *et al*. G196 epitope tag system: a novel monoclonal antibody, G196, recognizes the small, soluble peptide DLVPR with high affinity. *Sci. Rep.* **7**, 43480; doi: 10.1038/srep43480 (2017).

**Publisher's note:** Springer Nature remains neutral with regard to jurisdictional claims in published maps and institutional affiliations.

## Supplementary Material

Supplementary Information

## Figures and Tables

**Table 1:** Crystal parameters, data collection and structure refinement.

Data Set	Protein
Resolution range (Å)	50.0–2.00
Space group	*P*2_1_2_1_2_1_
Unit cell dimensions (Å)	*a* = 38.65, *b* = 82.27, *c* = 127.31
Reflections (Measured/Unique)	132,646/26,685
Completeness (Overall/Outer Shell,%)	94.3/82.7
*R*merge (Overall/Outer Shell, %)^a^	4.8/27.9
Mean < *I*>/<s(*I*) >(Overall)	18.7
Redundancy(Overall)	5.1
**Refinement Statistics**	
Resolution range (Å)	20.0–2.00
*R*-factor (%)^b^/free *R*-factor (%)	21.1/28.1
Number of water moleculaes	108
Ramachandran plot	
residues in most favorable regions (%)	95.0
residues in allowed regions (%)	4.5
residues in outlier regions (%)	0.5

Values in outer shell are for the highest shell with a resolution of 2.03–2.00 Å.

^a^*R*merge = S | *I*_*i*_ - < *I* > |/S | *I*_*i*_ |. where *I*_*i*_ is the intensity of an observation and <*I* > in the mean value for that reflection and the summations are over all reflections. free R-factor was calculated with 5% of the data.

^b^*R*-facter = S _*h*_||*F*o(*h*)| - |*F*c(*h*)||/S_*h*_*F*o(*h*), where *F*o and *F*c are the observed and calculated structure factor amplitudes, respectively.
