# Supplementary material for: G196 epitope tag system: a novel monoclonal antibody, G196, recognizes the small, soluble peptide DLVPR with high affinity
Source: Sci Rep. 2017 Mar 7;7:43480. doi: 10.1038/srep43480 (PMC5339894; doi:10.1038/srep43480)
Supplement: Supplementary Information [file srep43480-s1.pdf]

Supplementary Information

G196 epitope tag system: a novel monoclonal antibody, G196, recognizes the small, soluble peptide DLVPR with high affinity

Kasumi Tatsumi[1,2], Gyosuke Sakashita[1], Yuko Nariai[1], Kosuke Okazaki[1], Hiroaki Kato[1], Eiji Obayashi[1], Hisashi Yoshida[3], Kanako Sugiyama[3], Sam-Yong Park[3, 4], Joji Sekine[2] and Takeshi Urano[1]*

[1]Department of Biochemistry, Shimane University School of Medicine, Izumo, Japan
[2]Department of Oral and Maxillofacial Surgery, Shimane University School of Medicine, Izumo, Japan
[3]Drug Design Group, Kanagawa Academy of Science and Technology, Kawasaki, Japan
[4]Protein Design Laboratory, Yokohama City University, Tsurumi, Japan

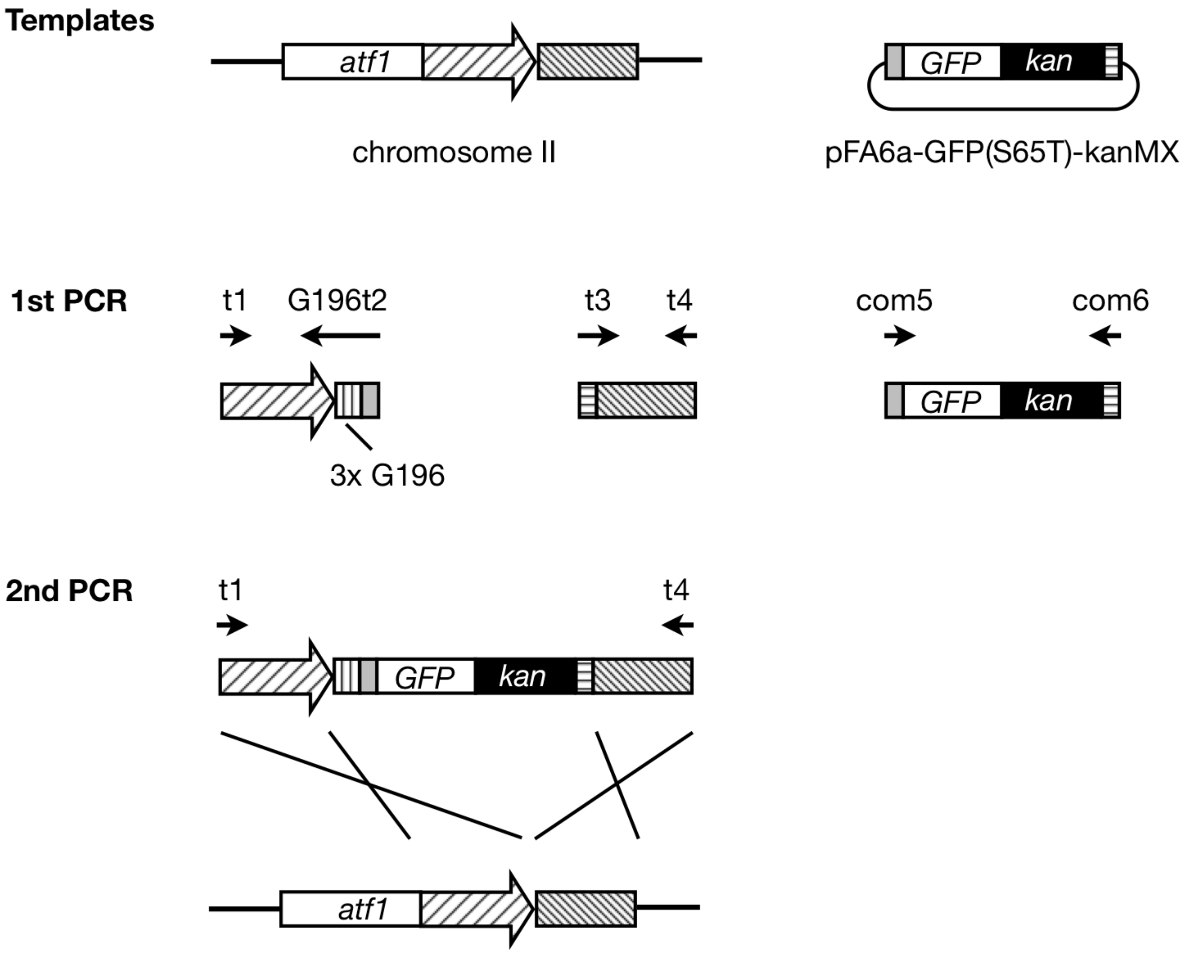

Supplementary Figure 1. Genetic manipulation of C-terminal tagging of the *atf1* gene

C-terminal tagging of the *atf1* gene was accomplished using the G196t2 primer. This primer contains a sequence homologous to a portion of the *atf1* coding sequence, a sequence for 3×G196-tag, and a sequence homologous to another primer, com5. In the first PCR step, two sequences flanking the translational stop codon of the *atf1* gene were amplified with the primers t1 and G196t2, and with the primers t3 and t4, using the genomic DNA as a template. The GFP-kanMX cassette was also amplified with the primers com5 and com6, using pFA6a-GFP(S65T)-kanMX as a template. The three PCR products were fused together in the second PCR step with the primers t1 and t4 to produce a DNA fragment for homologous recombination.

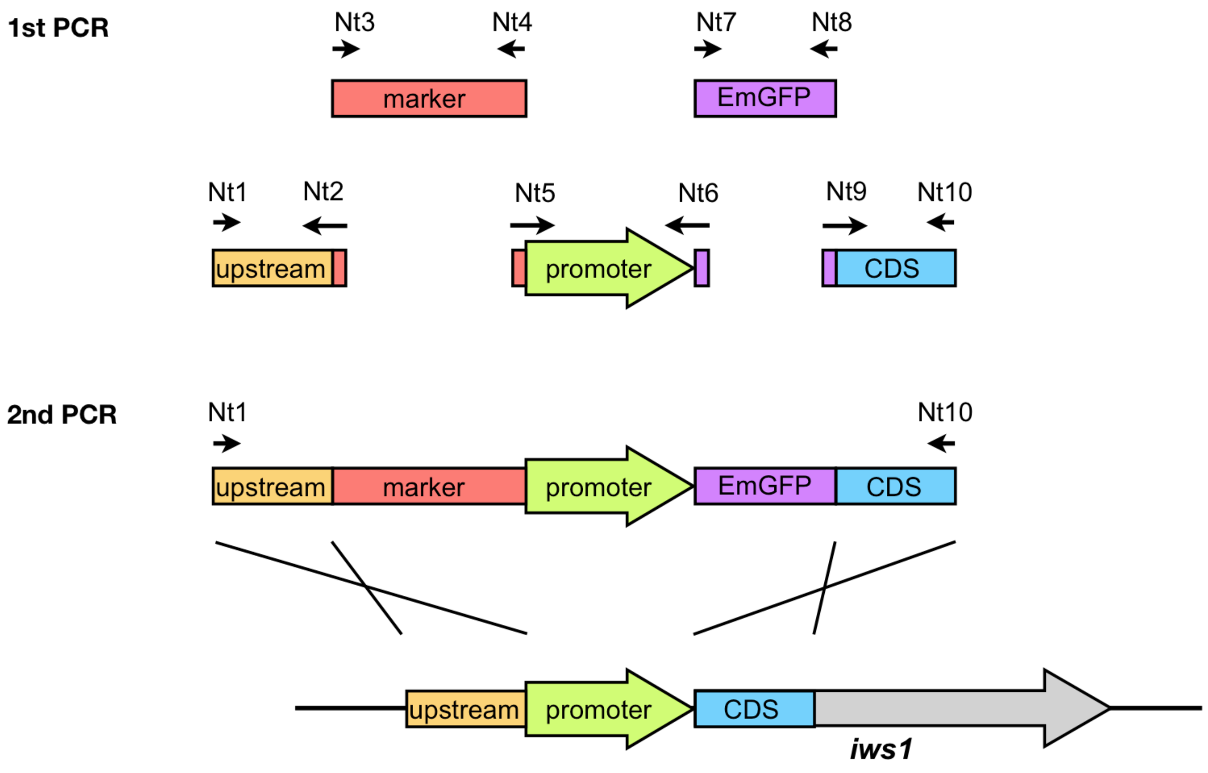

Supplementary Figure 2. Genetic manipulation of N-terminal tagging of the *iws1* gene

N-terminal tagging of the *iws1* gene was accomplished by amplifying the kanMX cassette from pFA6a-kanMX with the primers Nt3 and Nt4 in the first PCR step. The EmGFP coding sequence of EmGFP/pCDNA3.1-neo was amplified with the primers Nt7 and Nt8. We also amplified three sequences from the *iws1* locus: one homologous to the upstream region of the *iws1* promoter (upstream), one homologous to the promoter (promoter), and one homologous to a portion of the *iws1* coding region (CDS). The three sequences were amplified as follows: upstream sequence using the primers Nt1 and Nt2, the promoter sequence using Nt5 and NT6, and the CDS sequence using Nt9 and Nt10. The Nt2, Nt5, Nt6 and Nt9 sequences contained a sequence homologous to Nt3, Nt4, Nt7 and Nt8, respectively. The five fragments produced in the first PCR step were fused together in the second PCR step with the primers Nt1 and Nt10 to produce a DNA fragment for homologous recombination.

Supplementary Table1. DLVPR-containing proteins

on all UniProtKB/Swiss-Prot (release 2016_08 of 07-Sep-16: 551987 entries) database sequences human_taxid:9606

	UniProt ID	Entry name	Protein names	Position(S)	Length
1	O75752	B3GL1_HUMAN	UDP-GalNAc:beta-1,3-N-acetylgalactosaminyltransferase 1 (Beta-1,3-GalNAc-T1) (EC 2.4.1.79) (Beta-1,3-galactosyltransferase 3)	248 - 252	331
2	Q9Y4D2	DGLA_HUMAN	Sn1-specific diacylglycerol lipase alpha (DGL-alpha) (EC 3.1.1.-) (Neural stem cell-derived dendrite regulator)	524 - 528	1042
3	Q8IZD9	DOCK3_HUMAN	Dedicator of cytokinesis protein 3 (Modifier of cell adhesion) (Presenilin-binding protein) (PBP)	160 - 164	2030
4	Q8N1I0	DOCK4_HUMAN	Dedicator of cytokinesis protein 4	160 - 164	1966
5	P78414	IRX1_HUMAN	Iroquois-class homeodomain protein IRX-1 (Homeodomain protein IRXA1) (Iroquois homeobox protein 1)	440 - 444	480
6	Q5VU65	P210L_HUMAN	Nuclear pore membrane glycoprotein 210-like (Nucleoporin 210 kDa-like) (Nucleoporin Nup210-like)	1225 - 1229	1888
7	Q9Y5F3	PCDB1_HUMAN	Protocadherin beta-1 (PCDH-beta-1)	575 - 579	818
8	O75192	PX11A_HUMAN	Peroxisomal membrane protein 11A (HsPEX11p) (28 kDa peroxisomal integral membrane protein) (PMP28) (Peroxin-11A)	79 - 83	247
9	P0CAT3	TLXNB_HUMAN	Putative TLX1 neighbor protein (TLX1 divergent gene protein)	91 - 95	122
10	Q14669	TRIPC_HUMAN	E3 ubiquitin-protein ligase TRIP12 (EC 6.3.2.-) (E3 ubiquitin-protein ligase for Arf) (ULF) (Thyroid receptor-interacting protein 12)	715 - 719	1992
11	Q9P243	ZFAT_HUMAN	Zinc finger protein ZFAT (Zinc finger gene in AITD susceptibility region) (Zinc finger protein 406)	237 - 241	1243

Supplementary Table 2. Primers used in the study

Target	Name	Sequence
6P-11	6P-11-S	5′-gatcgggagaccatcctccaaaatcggatctggttccgcgtggatccccgg-3′
	6P-11-AS	5′-aattccggggatccacgcggaaccagatccgattttggaggatggtctccc-3′
6P-12	6P-12-S	5′-gatcgtcggatctggttccgcgtggatccccgg-3′
	6P-12-AS	5′-aattccggggatccacgcggaaccagatccgac-3′
6P-13	6P-13-S	5′-gatcgggagaccatcctccaaaatcggatctggttccgcgtg-3′
	6P-13-AS	5′-aattcacgcggaaccagatccgattttggaggatggtctccc-3′
6P-14	6P-14-S	5′-gatcgtcggatctggttccgcgtg-3′
	6P-14-AS	5′-aattcacgcggaaccagatccgac-3′
6P-15	6P-15-S	5′-gatcggatctggttccgcgtg-3′
	6P-15-AS	5′-aattcacgcggaaccagatcc-3′
6P-16	6P-16-S	5′-gatcgtcggatctggttccgg-3′
	6P-16-AS	5′-aattccggaaccagatccgac-3′
6P-17	6P-17-S	5′-gatcggatctggttccgg-3′
	6P-17-AS	5′-aattccggaaccagatcc-3′
6P-19	6P-19-S	5′-gatcggatctggttg-3′
	6P-19-AS	5′-aattcaaccagatcc-3′
6P-24	6P-24-S	5′-gatcggctctggttccgcgtg-3′
	6P-24-AS	5′-aattcacgcggaaccagagcc-3′
6P-25	6P-25-S	5′-gatcggatgctgttccgcgtg-3′
	6P-25-AS	5′-aattcacgcggaacagcatcc-3′
6P-26	6P-26-S	5′-gatcggatctggctccgcgtg-3′
	6P-26-AS	5′-aattcacgcggagccagatcc-3′
6P-27	6P-27-S	5′-gatcggatctggttgcccgtg-3′
	6P-27-AS	5′-aattcacgggcaaccagatcc-3′
6P-28	6P-28-S	5′-gatcggatctggttccggctg-3′
	6P-28-AS	5′-aattcagccggaaccagatcc-3′
6P-29	6P-29-S	5′-gatcggctgctgctgccgctg-3′
	6P-29-AS	5′-aattcagcggcagcagcagcc-3′
6P-30	6P-30-S	5′-gatcggaactggttccgcgtg-3′
	6P-30-AS	5′-aattcacgcggaaccagttcc-3′
6P-31	6P-31-S	5′-gatcggatctggttccgaagg-3′
	6P-31-AS	5′-aattccttcggaaccagatcc-3′
V_H	VH1-1S	5′-ggggatccaggtsmarctgcagsagtcwgg-3′
	IgG2-1AS	5’-gggaattccttgaccaggcatcctagagtca-3’
L_H	VK-1S	5′-ggggatccgayattgtgmtsacmcarwctmca-3′
	CK-2AS	5′-gggaattcgaagatggatacagttggtgc-3’

s=g+c、m=a+c、r=a+g、w=a+t, y=c+t

Primers used in the study for yeast genetic manipulations

**Target**	**Name**	**Sequence**
Atf1	t1	5′-tgccaacggcaaattcgatg-3′
	G196t2	5′-tcgacctgcagcgtacgaGGACCCTCC*CCTGGGGACCAAGTC*AGAGCCACC*ACGTGGTACGAGATC*GCTTCCCCC*GCGCGGCACTAGGTC*ACTCCCGCCgtaccctaaattgattctttgag-3′
	t3	5′-TAAACGAGCTCGAATTCATCGAT-aaggtctcagtctgtgatgg-3′
	t4	5′-tactccacaatacactaagac-3′
pFA6a	com5	5′-TCGTACGCTGCAGGTCGA-3′
	com6	5′-ATCGATGAATTCGAGCTCGTTTA-3′
Iws1	Nt1	5′-agaaacgaccaacatgaaatc-3′
	Nt2	5′-GACGAGGCAAGCTAAACAGATatggatggttattgtacgtcg-3′
	Nt3	5′-ATCTGTTTAGCTTGCCTCGTC-3′
	Nt4	5′-GGCGTTAGTATCGAATCGAC-3′
	Nt5	5′-GTCGATTCGATACTAACGCCgcacaaaagccaacacttgg-3′
	Nt6	5′-CCTCGCCCTTGCTCACCATctttggagaatcagaaatttg-3′
	Nt7	5′-ATGGTGAGCAAGGGCGAGG-3′
	Nt8	5′-CTTGTACAGCTCGTCCATGC-3′
	Nt9	5′-GCATGGACGAGCTGTACAAGGGTGGAGGCGGGtcagaagaagaaaaggctgag-3′
	Nt10	5′-cgcttctttgttcgagttgg-3′

Sequences homologous to the genome are written in small letters. Underlined letters represent codons for linker amino acids: a Gly-Gly-Gly-Gly peptide for N-terminal tagging of iws1, Gly-Gly-Ser peptides in the 3xG196 epitope tag.
Sequences encoding the G196 epitope DLVPR are written in italic.

Supplementary Table 3. Fission yeast strains used in this study

Strain #	Genotype	Reference
HKM-1102	*h*$^{+}$, *ade6-DN/N, leu1-32, ura4-DS/E, imr1L::ura4*$^{+}$, *otr1R::ade6*$^{+}$	Ref. #1
HKM-1984	*h*$^{-}$, *ade6-DN/N, ura4-DS/E, imr1L::ura4*$^{+}$, *otr1R::ade6*$^{+}$, *kanMX-GFP-Iws1*	This study
HKM-2011	*h*$^{+}$, *ade6-DN/N, leu1-32, ura4-DS/E, imr1L::ura4*$^{+}$, *otr1R::ade6*$^{+}$, *atf1-G196-GFP-kanMX*	This study

Ref. #1
Kato, H. *et al.* Spt6 prevents transcription-coupled loss of posttranslationally modified histone H3. *Sci. Rep.* **3**, 2186 (2013).
